# IL-1β alters the virulence of uropathogenic *Escherichia coli*

**DOI:** 10.1038/s41598-025-26055-4

**Published:** 2025-10-29

**Authors:** Fatma Kalaycı-Yüksek, Rongrong Wu, Ignacio Rangel, Isak Demirel

**Affiliations:** 1 Faculty of Medicine, Department of Medical Microbiology, Istanbul Yeni Yüzyıl University, Istanbul, Turkey; 2 School of Medical Sciences, Örebro University, Campus USÖ, Örebro, SE-701 82 Sweden

**Keywords:** Uropathogenic *E. coli*, IL-1β, *C. elegans*, Colonization, Cross-kingdom interaction, Immunology, Microbiology

## Abstract

Uropathogenic *Escherichia coli* (UPEC) is the leading cause of urinary tract infection (UTI). While the role of UPEC in triggering host immune responses is well studied, less is known about how host-derived cytokines influence UPEC virulence. The aim of this study was to investigate how the pro-inflammatory cytokine IL-1β affects UPEC virulence, metabolism, and host-pathogen interactions. Using the CFT073 strain, we found that IL-1β exposure induced a rapid metabolic shift characterized by decreased oxygen consumption rate (OCR) and increased extracellular acidification rate (ECAR), indicating a transition from respiration to fermentative metabolism. Microarray analysis confirmed the upregulation of fermentative (*hyc*) and antioxidant (*katG*,* ahpF*,* grxA*) genes, along with increased purine biosynthesis, supporting a metabolic state favouring stress resistance and proliferation. IL-1β also increased *fimH* and *papC* gene expression, leading to increased adhesion and invasion of bladder epithelial cells. Furthermore, IL-1β-stimulated CFT073 suppressed the gene expression of several innate immune related genes in a *C. elegans* infection model. Taken together, our findings suggest that IL-1β induces a virulence-enhancing metabolic change in UPEC, which could enhance its persistence and colonization of the urinary tract. This cross-kingdom signaling may have implications for infection dynamics during a UTI.

**Supplementary Information:**

The online version contains supplementary material available at 10.1038/s41598-025-26055-4.

## Introduction

Uropathogenic *Escherichia coli* (UPEC) is the predominant cause of urinary tract infection (UTI)^1^. UPEC possesses a wide array of virulence factors that enable it to colonize, persist, and damage the host urinary tract. These include adhesins such as type-1-fimbriae and P-fimbriae, toxins, siderophores and immune modulators that contribute to host tissue invasion, iron acquisition and immune evasion. UPEC can invade bladder epithelial cells, replicate intracellularly, and form intracellular bacterial communities (IBCs), which are protected from immune detection and antimicrobial treatment^2–6^. In addition, UPEC can modulate host immune responses, including cytokine production and epithelial barrier integrity, further contributing to chronic infection and recurrence^7–9^.

UTI is an infection characterized by a strong innate immune response. A key component of this response is the release of pro-inflammatory cytokines, which include interleukin-6 (IL-6), interleukin-8 (IL-8) and interleukin-1β (IL-1β). These cytokines are rapidly induced upon UPEC adhesion to uroepithelial cells and are essential for the recruitment and activation of neutrophils and macrophages at the site of infection^10–18^. IL-1β plays a particularly central role in coordinating early immune responses in the urinary tract^18,19^. IL-1β is produced as an inactive pro-cytokine and is activated through inflammasome-mediated proteolytic cleavage, leading to its release during epithelial damage or infection^18,20^. IL-1β not only promotes immune cell infiltration but also drives epithelial remodelling and antimicrobial peptide expression^21,22^. However, while the host uses IL-1β as a defence mechanism, our recent findings suggest that UPEC can sense IL-1β as a signal to adapt and enhance its virulence^23^. This cross-kingdom interaction is supported by accumulating evidence showing that IL-1β exposure can enhance bacterial adhesion, biofilm formation, immune evasion, and metabolic reprogramming in pathogens such as *Pseudomonas aeruginosa*, *Staphylococcus aureus* and UPEC^17,23–25^. Despite this emerging knowledge, the specific effects of IL-1β on UPEC host-pathogen dynamics remain poorly understood. To address this knowledge gap, we recently investigated how exposure to pro-inflammatory cytokines affect the virulence traits of the UPEC strain CFT073. Our findings showed that IL-1β, along with other cytokines, significantly increased UPEC growth in a concentration-dependent manner. Notably, IL-1β exposure upregulated genes involved in iron acquisition and gluconeogenesis, while simultaneously reducing biofilm formation and hemolytic activity. Interestingly, despite this reduction in hemolytic activity, the overall virulence of UPEC, assessed using a *Caenorhabditis elegans (C. elegans)* infection model, was increased in the presence of IL-1β ^23^. Hence, suggesting a complex modulation of bacterial behaviour that favours persistence and host colonization. Despite these initial findings, we still don’t know how IL-1β affects UPEC at a transcriptomic or metabolic level. Given the increasing recognition that pathogens can sense and respond to host-derived cytokines as environmental signals^25^, it is important to investigate how this crosstalk influences bacterial adaptation and host defence regulation. The aim of this study was to investigate how IL-1β affects UPEC virulence by examining global transcriptional and metabolic changes, as well as its impact on host-pathogen interactions in vivo, using *C. elegans* as a model.

## Materials and methods

### Bacteria and bladder epithelial cells

The uropathogenic *E. coli* strain CFT073 was a kind gift from Professor Harry Mobley at University of Michigan Medical School, MI, USA. CFT073 was kept on tryptic soy agar (TSA) and grown in Lysogeny broth (Difco Laboratories, Detroit, MI, USA) overnight on shaking (150 rpm) at 37 °C prior to experiments. Human bladder epithelial cell line HBLAK (CELLnTEC Advanced Cell Systems AG, Bern, Switzerland), spontaneously immortalized, was grown in Gibco™ EpiLife™ Medium, with 60 µM calcium, supplemented with Gibco™ Human Keratinocyte Growth Supplement (HKGS, ThermoFisher Scientific, MA, USA) at 37 °C, 5% CO_2_ atmosphere. The complete medium was supplemented with 1 mM CaCl_2_ 24 h prior to experiments.

### Microarray analysis

CFT073 (1*10^6^ CFU/ml) was grown in minimal salt medium (MSM, 1.3% [wt/vol] Na_2_HPO_4_, 0.3% KH_2_PO_4_, 0.05% NaCl, and 0.1% NH_4_Cl supplemented with 20 mM glucose, 2 mM MgSO_4_, 100 µM CaCl_2_, and 0.25% Casamino Acids) with or without the presence of 0.5 ng/ml IL-1β (I9401, Sigma-Aldrich, St. Louis, MO, USA) for 6 hours in a 96-well plate. Total RNA was isolated from the bacteria with the RNeasy Mini Kit (Qiagen Technologies, Hilden, Germany) according to manufacturer instructions. DNA contamination was removed by DNase digestion (TURBO DNase, Life technologies, MA, USA) according to manufacturer instructions. RNA concentration and purity were measured using a Nano-Drop ND-1000 Spectrophotometer (Nano-Drop Technology Inc., Wilmington, DE, USA). RNA quality was also evaluated using a Agilent 2100 Bioanalyzer (Agilent Technologies, Palo Alto, CA, USA) according to manufacturer instructions. All samples had RNA integrity number (RIN) values above 9. Total RNA from three biological replicates was used to prepare labeled cRNA with Low Input Quick Amp WT Labeling Kits (Agilent) according to manufacturer instructions. Labeled cRNA samples were hybridized in a G2545A hybridization oven (Agilent) onto Agilent SurePrint G3 *E. coli* Gene Expression 8 × 15k (Agilent Technologies) glass arrays according to manufacturer instructions and subsequently scanned with a G2565 CA array laser scanner (Agilent Technologies). Image analysis and data extraction was performed with Feature Extraction Software (version 10.7.3.1, Agilent Technologies).

### RNA isolation, cDNA synthesis and real time-qPCR

CFT073 (1*10^6^ CFU/ml) was grown in MSM with or without the presence of IL-1β (0.5 or 40 ng/ml) in a 96-well plate for 24 h at 37 °C. The bacteria were treated with RNAlater (Sigma-Aldrich) prior to isolation of total RNA using the E.Z.N.A ^®^ Total RNA Kit I (Omega Bio-tek, Inc., Norcross, GA, USA) according to manufacturer instructions. DNA contamination was removed by DNase digestion (TURBO DNase, Life technologies) according to manufacturer instructions. A total of 100 ng was reverse transcribed to cDNA using the High-Capacity cDNA Reverse Transcription Kit for single-stranded cDNA synthesis (Applied Biosystems, CA, USA). Maxima SYBR Green qPCR Master Mix (ThermoFisher Scientific, MA, USA) was used for the real time-qPCR. Each reaction contained 5 ng of template cDNA and 250 nM of each primer (Table 1, Eurofins MWG Synthesis GmbH, Ebersberg, Munich, Germany) was used in the real time-qPCR. A CFX96 Touch™Real-Time PCR Detection System (Biorad, CA, USA) was used for the amplification using the following protocol: initial denaturation at 95 °C for 10 min, 40 cycles of denaturation at 95 °C for 15 s followed by annealing at 60 °C for 30 s and extension at 72 °C for 30 s. The PCR was followed by a dissociation curve analysis between 60 and 95 °C. The Ct values were analyzed by the ΔΔCt method and normalized to the endogenous control *gapA* (glyceraldehyde 3-phosphate dehydrogenase A). Fold difference was calculated as 2^− ΔΔCt^.

**Table 1:** Primers used in the real-time qPCR.

*Gene*	*Function*	*Forward primer (5’–3’)*	*Reverse primer (5’–3’)*
*abf-2*	Microbicidal activity	CCGTTCCCTTTTCCTTGCAC	GACGACCGCTTCGTTTCTTG
*ape-1*	Apoptosis	GTTTGGTGATAGTCTAGACG	TGTTGTGGTATCACTACCTAATACC
*bar1*	Wnt signaling	CCTAATTTGCACGCTACGGC	TCGGCATGATCGGAATGGAG
*cep-1*	Apoptosis	AGGGCACGATTCAGTGTTG	TTCATCGCTTCCTGGATGC
*clec-60*	Antimicrobial defence	TGTCTGCATTCTTCCAGTCG	CCCATACCCAGACACCTTTG
*lys-7*	Antimicrobial response	ATTCAGGTCACTTCGCCAAC	ATCCGGTCGTGATCTGATTC
*tir-1*:	Innate immune response	TTGGGTGCACAAAGAGCTGA	GGTCGGTGTCGTTCTGTTCA
*wah-1*	Apoptosis	GCTGATGCTGTCGAGGAGA	TGGTGGTGTTCTCTTCTGTAGA
*Y45F10D.4 (FES)*	Fes, an iron-sulfur cluster assembly enzyme (endogenous control)	CTGGCAACCGAATGGATTA	CTTCTTAGTCTGCTTCTTCTGATA
*fimH*	Type-1 fimbriae	GTGCCAATTCCTCTTACCGTT	TGGAATAATCGTACCGTTGCG
*papC*	P fimbriae	GTGGCAGTATGAGTAATGACCGTTA	TATCCTTTCTGCAGGGATGCAATA
*grxA*	Glutaredoxin1 redox coenzyme for glutathione-dependent ribonucleotide reductase	ATGAGCGCGATGATTTCCAG	CTCTTTCACCCATGCAGCAA
*purL*	Phosphoribosylformyl-glycineamide synthetase	ACCTCGACTTTGCTTCCGTA	GCGCAGTTCAAATTTACCGC
*rlpB*	A minor lipoprotein	GCCAAAGTCTTCCGTTCGTT	GTTGTGGTCGACGTCTGTTC
*nirB*	Nitrite reductase	GAAGAACTGCTGGCGAAACA	CTTTCTGGATTGTGCGGAGG
*gapA*	Glyceraldehyde-3-phosphate dehydrogenase (endogenous control)	AAGTTGGTGTTGACGTTG	AGCGCCTTTAACGAACATCG

### Invasion and colonization assay

CFT073 (1*10^6^ CFU/ml) was grown in MSM with or without the presence of IL-1β (0.5 or 40 ng/ml) for 24 h in 37 °C. IL-1β was then washed away from the bacteria with PBS. Intracellular bacterial invasion was assessed by stimulating the human bladder epithelial cell line HBLAK with the bacterial at MOI 10 for 2 h at 37 °C with 5% CO_2_. The cells were washed with PBS after stimulation and the culturing medium was replaced with EpiLife™ Medium complemented with 100 µg/ml gentamicin for an additional 2 h. The cells were thereafter washed, lysed with 0.1% Triton-X 100 in PBS, plated on TSA plates and grown overnight at 37 °C followed by CFU counting. The data is presented as % of CFT073 ^21^.

CFT073 (1*10^6^ CFU/ml) was grown in MSM with or without the presence of IL-1β (0.5 or 40 ng/ml) for 24 h in 37 °C. HBLAK were infected with CFT073 (eGFP, pLMB449 plasmid) at MOI 10 for 4 h at 37 °C with 5% CO_2_. The assay used to evaluate bacterial colonization (adherent and intracellular bacteria) of HBLAK cells. The HBLAK cells were then washed ten times with PBS, and the fluorescence (eGFP) was measured using the Cytation 3 plate reader. Colonization is presented as mean fluorescence intensity (MFI)^21^.

### Measurement of extracellular oxygen and acid flux

The oxygen consumption rate (OCR) and extracellular acidification rate (ECAR) of CFT073 were measured using a Seahorse XF HS Mini Analyzer (Agilent Technologies, Didcot, UK). CFT073 was first cultured overnight in LB broth at 37 °C with shaking (150 rpm). The culture was centrifuged at 4000 × *g* for 10 min, and the bacterial pellet was resuspended in MSM. A suspension of CFT073 (1*10^7^ CFU/ml) was then transferred to an XF HS miniplate (200 µl final volume per well). The bacteria were subsequently stimulated with IL-1β (0.5 or 40 ng/ml) or left unstimulated and centrifuged at 3000 × *g* for 10 min. The first OCR and ECAR reading was recorded 30 min after stimulation and continued for 125 min at 37 °C in the analyzer.

### Motility assay

CFT073 was grown in MSM with or without the presence of IL-1β (0.5 or 40 ng/ml) for 24 h in 37 °C. A 10 µl suspension of CFT073 at 1*10^10^ CFU/ml was then stabbed into the middle of a soft agar plate (2.5 g/l agar, 10 g/l tryptone, 5 g/l NaCl) and incubated for 16 h at 30 °C. Bacterial motility was evaluated by measuring the diameter of the migration zone^26^.

### Cytokine release

CFT073 (1*10^6^ CFU/ml) was grown in MSM with or without the presence of IL-1β (0.5 or 40 ng/ml) for 24 h at 37 °C. IL-1β was then washed away from the bacteria with PBS. Cytokine release was assessed by stimulating the human bladder epithelial cell line HBLAK with the bacterial at MOI 10 for 4 h at 37 °C with 5% CO_2_. The cytokines were measured with the IL-1β, IL-6 and IL-8 kits (ELISA MAX Deluxe Sets, BioLegend, San Diego, CA, United States) according to the manufacturer’s instructions.

### *C. elegans* gene expression

The Bristol wild type N2 strain of *C. elegans* (Caenorhabditis Genetics Center, University of Minnesota, USA) was maintained on nematode growth medium plates seeded with a lawn of *E. coli* OP50 at 21 °C. Prior to experiments, *C. elegans* were alkaline/hypochlorite-synchronized (0.25 M NaOH, 1% HOCl) and maintained on nematode growth medium plates for 48 h at 21 °C to reach L4 stage. CFT073 was grown in MSM with or without the presence of IL-1β (0.5 or 40 ng/ml) for 24 h at 37 °C. IL-1β was then washed away from the bacteria with PBS. Age synchronized L4 worms were washed with MSM and 200 worms were then transferred to respective well of bacteria (5*10^8^ CFU/ml). After the addition of the worm to the wells, *C. elegans* and the bacteria were incubated together for 5 h at 21 °C. Total RNA was isolated by homogenizing the worms using a TissueLyser LT (Qiagen, Hilden, Germany) (0.5 mm beads, 2 cycles of 2 min at 30 Hz) followed by RNA isolation with the RNeasy plus mini kit (Qiagen). A total of 35 ng RNA was reverse transcribed to cDNA using the High-Capacity cDNA Reverse Transcription Kit for single-stranded cDNA synthesis (Applied Biosystems). Maxima SYBR Green qPCR Master Mix (ThermoFisher Scientific) was used for the real time-qPCR. Each reaction contained 1.75 ng of template cDNA and 250 nM of each primer (Table 1, Eurofins MWG Synthesis GmbH) was used in the real time-qPCR. A CFX96 Touch™Real-Time PCR Detection System (Biorad, ) was used for the amplification using the following protocol: initial denaturation at 95 °C for 10 min, 40 cycles of denaturation at 95 °C for 15 s followed by annealing at 60 °C for 30 s and extension at 72 °C for 30 s. The qPCR was followed by a dissociation curve analysis between 60 and 95 °C. The Ct values were analyzed by the ΔΔCt method and normalized to the endogenous control *Y45F10D.4 (FES)*. Fold difference was calculated as 2^− ΔΔCt^.

### Statistical analysis

Data are shown as mean ± standard error of the mean (SEM), n = number of independent experiments. Differences between groups were assessed by unpaired Student’s t-test. All microarray data analysis was performed using Gene Spring GX version 12.0 (Agilent Technologies) after per chip and gene 75th percentile shift normalization of samples. Differential expression between groups was analyzed with unpaired Student’s t-test. Given the exploratory nature of the study and the relatively small sample size, correction for multiple testing was not applied in order to avoid overlooking potentially biologically relevant genes. Instead, we applied a fold-change cutoff (≥ 1.5) together with pathway-level validation using GO enrichment and KEGG analysis to increase the robustness of the results. Fold change was set at ≥ 1.5 for significantly expressed entities (*p* < 0.05). GO enrichment and KEGG pathway analysis^27–29^ were conducted with STRING (version 10.5) and significance was set at *p* < 0.05.

## Results

### Global transcriptional changes induced by IL-1β stimulation

**Fig. 1:**
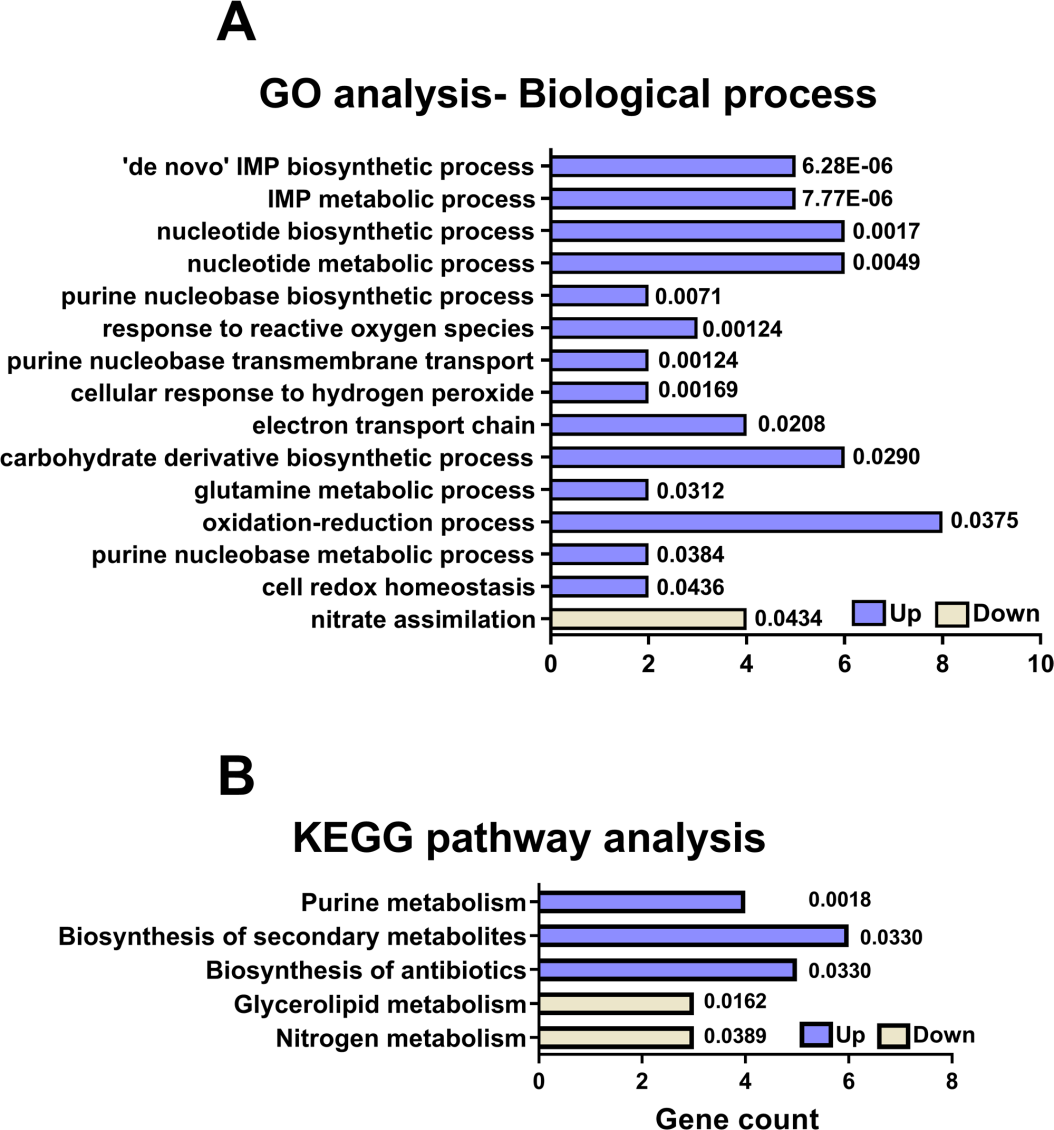
Gene ontology and KEGG pathway analysis. The enriched GO-biological processes (**A**) and KEGG pathways^27–29^ (**B**) in CFT073 after IL-1β stimulation for 6 h. Each enriched Gene ontology and KEGG pathway is presented with respective false discovery rate < 0.05.

Microarray profiling was performed on CFT073 following stimulation with IL-1β (0.5 ng/ml) compared to unstimulated CFT073. Differential expression analysis identified that 31 gene entities (Supplementary Table S1) were significantly upregulated, and 45 gene entities (Supplementary Table S2) were significantly downregulated upon IL-1β stimulation (*p* < 0.05) with at least a ≥ 1.5-fold change compared to unstimulated CFT073. Validation of the microarray data by RT-qPCR for *grxA*, *purL*, *rlpB*, and *nirB* confirmed the same significant changes in gene expression (Fig. S1). Gene ontology (GO) analysis was performed on significantly altered genes following IL-1β stimulation of CFT073. In total, 14 GO were enriched among upregulated genes (Supplementary Table S3) and 1 (Supplementary Table S4) among downregulated genes. These were mainly related to purine biosynthesis, oxidative stress response, and nucleotide metabolism (Fig. 1A). KEGG pathway analysis identified 3 enriched pathways among upregulated genes (Supplementary Table S5) and 2 (Supplementary Table S6) among downregulated genes. Upregulated pathways included purine metabolism and biosynthesis of secondary metabolites, while downregulated ones were related to glycerolipid and nitrogen metabolism (Fig. 1B).

### IL-1β alters the metabolic activity of CFT073

**Fig. 2:**
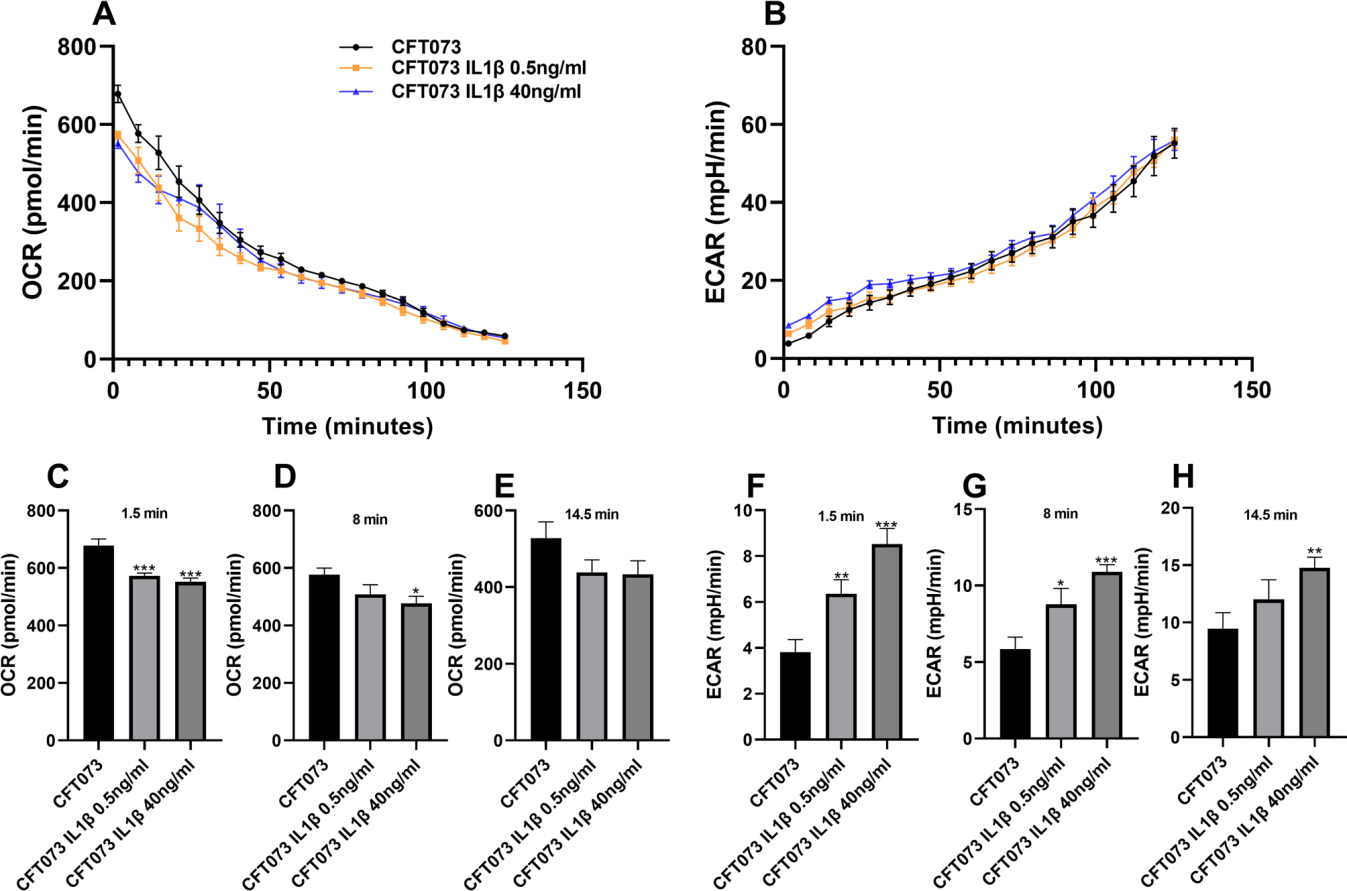
IL-1β stimulation alters CFT073 metabolism. Oxygen consumption rate (OCR, **A**, **C**, **D**, **E**) and extracellular acidification rate (ECAR, **B**, **F**, **G**, **H**) was measured in real time in CFT073 following IL-1β stimulation for 125 min. Data are presented as mean ± SEM (*n* = 8 independent experiments). Asterisks denote statistical significance compared to CFT073 (*p < 0.05, **p < 0.01, ***p < 0.001).

To evaluate the metabolic changes induced by IL-1β, the oxygen consumption rate (OCR) and extracellular acidification rate (ECAR) of CFT073 were measured following stimulation with IL-1β at 0.5 and 40 ng/ml. IL-1β stimulation induced a rapid decrease in OCR (Fig. 2A), with significantly reduced levels observed after 1.5 (Fig. 2C) and 8 min (Fig. 2D), but not after 14.5 min (Fig. 2E) compared to unstimulated CFT073. In contrast, ECAR measurements showed that IL-1β significantly increased the acidification rate of CFT073 at 1.5 min (Fig. 2F), 8 min (Fig. 2G) and after 14.5 min (Fig. 2H) compared to unstimulated CFT073 (Fig. 2B).

### IL-1β enhances adhesion, invasion, and fimbrial gene expression in CFT073

**Fig. 3:**
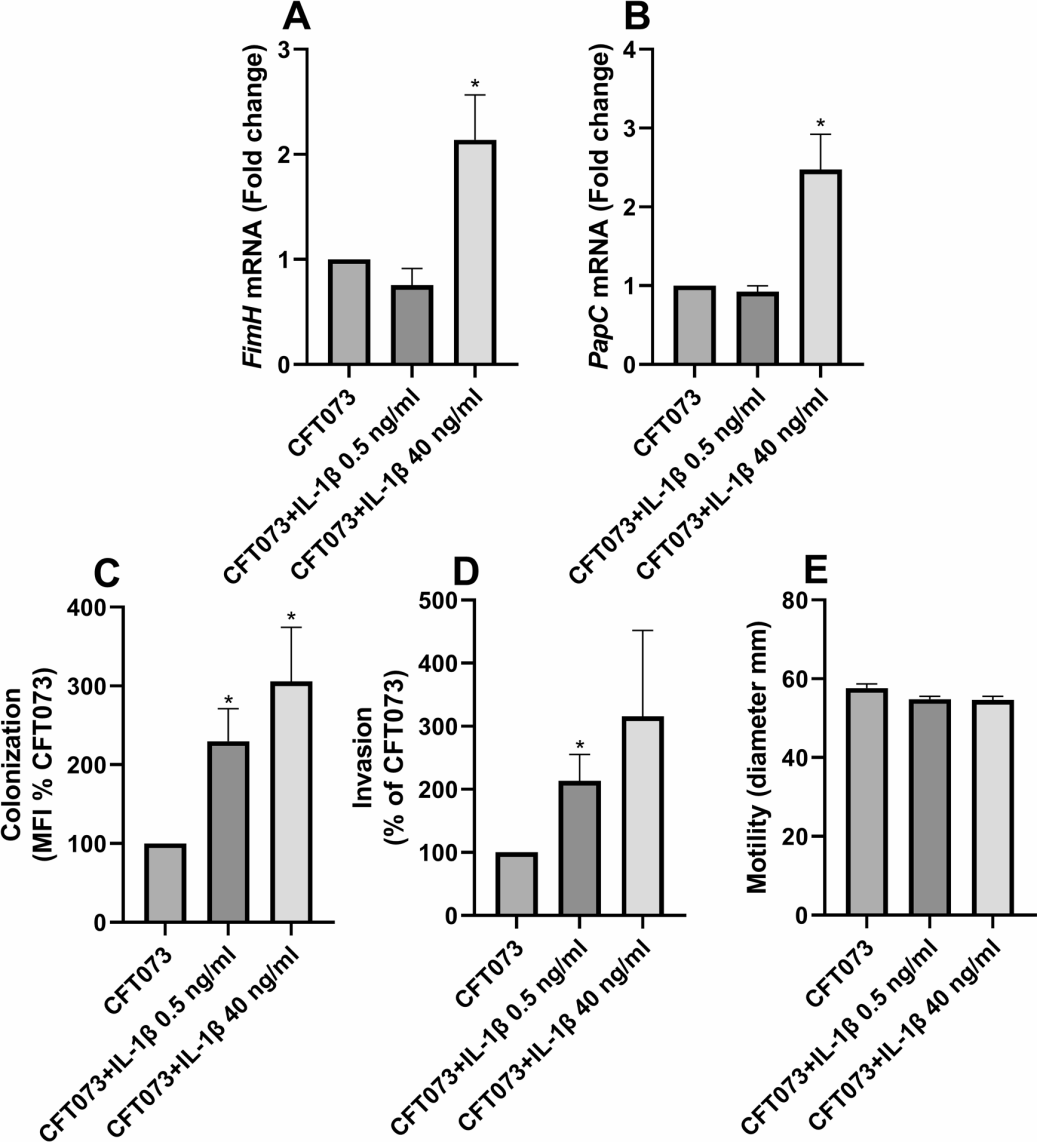
Increased bacterial colonization and invasion. Real time-qPCR analysis of *fimH* (**A**) and *papC* (**B**) mRNA expression in the presence or absence of IL-1β (0.5 or 40 ng/ml) after 24 h. Data are presented as mean ± SEM of *n* = 3–4 independent experiments. CFT073 colonization (**C**) and invasion (**D**) of bladder epithelial cells in the presence or absence of IL-1β (0.5 or 40 ng/ml) after 2 h (**C**) and 4 h (**D**). Colonization was quantified as mean fluorescence intensity (MFI) (**C**). Data are presented as mean ± SEM of *n* = 4–5 independent experiments. CFT073 motility assay in the presence or absence of IL-1β (0.5 or 40 ng/ml) after 16 h (**E**). Data are presented as mean ± SEM of *n* = 5 independent experiments. Asterisks denote statistical significance compared to CFT073 (*p < 0.05).

Next, we investigated whether IL-1β stimulation affected CFT073’s fimbriae gene expression and colonization capacity. Real-time qPCR analysis revealed that IL-1β significantly increased the gene expression of the fimbrial genes *fimH* and *papC* at 40 ng/ml compared to unstimulated CFT073 (Fig. 3A, B). To determine the functional relevance of these changes, we assessed bacterial colonization and invasion of human bladder epithelial cells. CFT073 stimulated with IL-1β significantly increased the colonization of bladder epithelial cells at both 0.5 and 40 ng/ml compared to unstimulated CFT073 (Fig. 3C). Furthermore, CFT073 invasion of bladder epithelial cells was significantly increased after IL-1β stimulation at 0.5 ng/ml, but not 40 ng/ml compared to unstimulated CFT073 (Fig. 3D). In addition, we also observed that IL-1β stimulation of CFT073 did not affect the motility of CFT073 (Fig. 3E).

### IL-1β-stimulated CFT073 did not increase the release of cytokines or chemokines

**Fig. 4:**
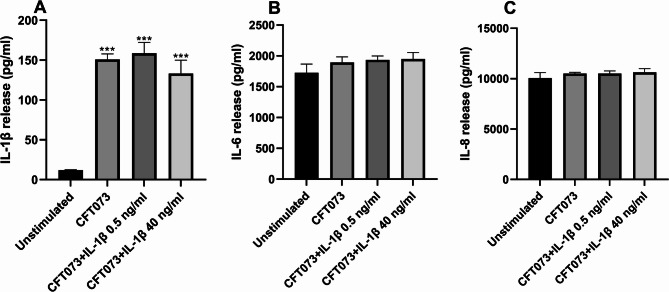
Cytokine release from bladder epithelial cells. Bladder epithelial cells we infected with CFT073 or IL-1β-stimulated CFT073 (0.5 or 40 ng/ml) at MOI 10 for 4 h followed by analysis of IL-1β (**A**), IL-6 (**B**) and IL-8 (**C**) release. Data are presented as mean ± SEM (*n* = 4 independent experiments). Asterisks denote statistical significance compared to unstimulated cells (***p < 0.001).

We proceeded with evaluating if IL-1β-stimulated CFT073 could influence cytokine or chemokine release from bladder epithelial cells. CFT073 alone and IL-1β-stimulated CFT073 significantly increased the release of IL-1β compared to unstimulated cells (Fig. 4A). However, this increased release was not altered by IL-1β-stimulated CFT073 compared to unstimulated CFT073 (Fig. 4A). No significant differences were observed in IL-6 (Fig. 4B) or IL-8 (Fig. 4C) release between IL-1β-stimulated CFT073 and unstimulated CFT073.

### IL-1β-stimulated CFT073 downregulates innate immune genes in *C. elegans*

**Fig. 5:**
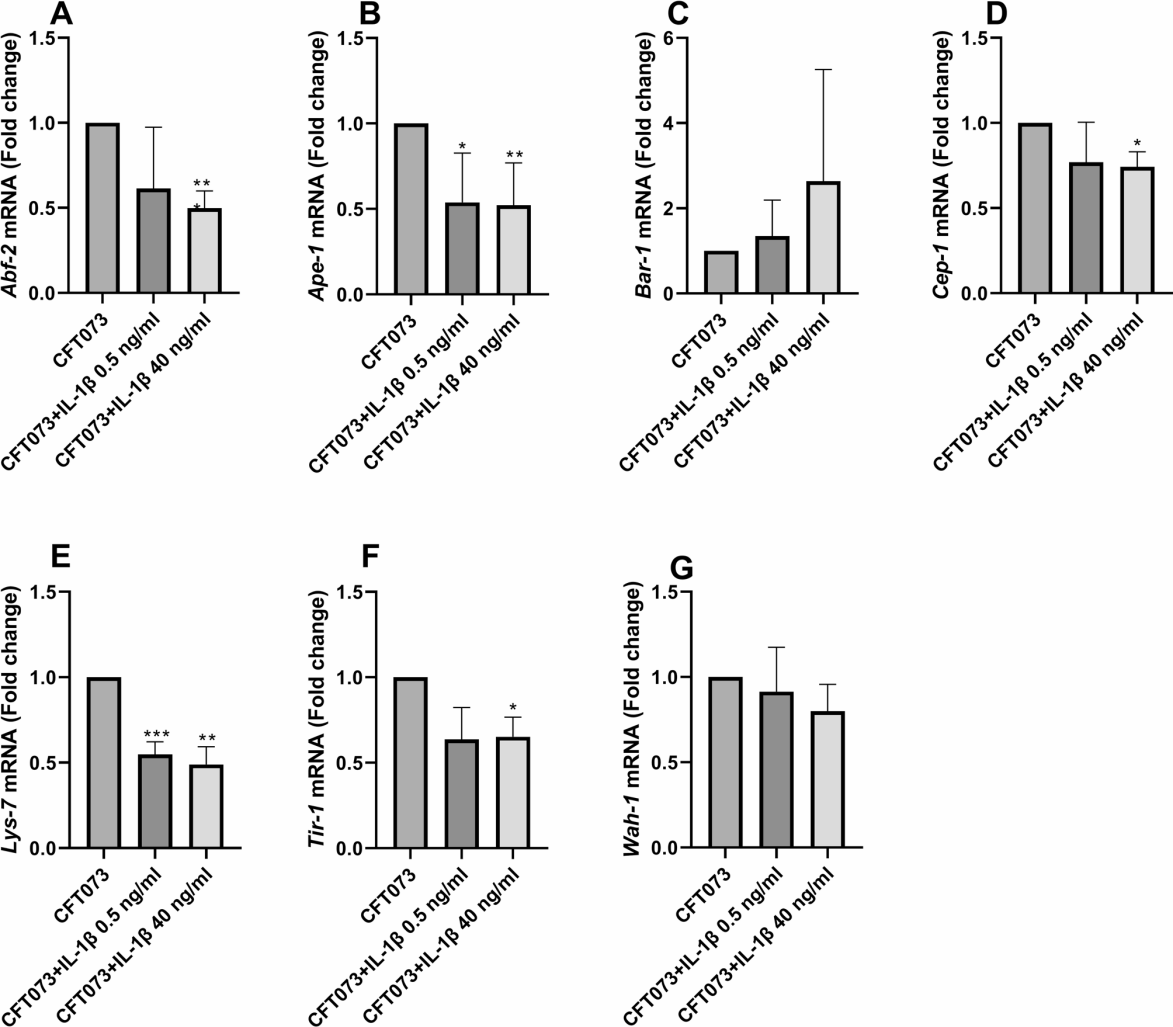
*C. elegans* mRNA expression. *C. elegans* worms we infected with CFT073 or IL-1β-stimulated CFT073 (0.5 or 40 ng/ml) for 5 h followed by analysis of gene expression of *Abf-2* (**A**), *Ape-1* (**B**), *Bar-1* (**C**), *Cep-1* (**D**), *Lys-7* (**E**), *Tir-1* (**F**) and *Wah-1* (**G**). Data are presented as mean ± SEM (*n* = 4 independent experiments). Asterisks denote statistical significance compared to CFT073 (*p < 0.05, **p < 0.01, ***p < 0.001).

To determine whether IL-1β stimulation of CFT073 affects host immune responses, we evaluated the expression of selected *C. elegans* immune and stress-related genes following CFT073 infection. Several *C. elegans* genes were significantly downregulated in worms infected with IL-1β-stimulated CFT073. We found that the gene expression of *abf-2* (Fig. 5A), *ape-1* (Fig. 5B), *Cep-1* (Fig. 5D), *lys-7* (Fig. 5E) and *Tir-1* (Fig. 5F) were significantly decreased after infection with IL-1β-stimulated CFT073 compared to unstimulated CFT073. However, we did not observe any significant changes in the expression of *bar-1* (Fig. 5C) or *wah-1* (Fig. 5G).

## Discussion

Understanding the interplay between UPEC and the host immune system is important, particularly considering the increasing antibiotic resistance that is limiting treatment options. In this study, we investigated the cross-kingdom impact of the pro-inflammatory cytokine IL-1β on UPEC virulence and metabolic adaptation. We chose to focus on IL-1β, as it is one of the most potent pro-inflammatory cytokines and it has been linked to dysregulated inflammation and to the severity of UTI^16,19,30–32^. We have previously also shown that IL-1β can enhance UPEC virulence traits, including increased bacterial growth^23^.

Our findings showed a rapid inverse relationship between OCR and ECAR following IL-1β stimulation. We found that changes in ECAR, but not in OCR, were dependent on the IL-1β concentration. This indicates a metabolic shift in CFT073 from oxygen-dependent respiration toward acidogenic, fermentative metabolism. In *E. coli*, this shift may be explained by an overflow metabolism^33^ or strict anaerobic fermentation. In both cases, pyruvate is converted by pyruvate-formate lyase into formate and acetyl-CoA. Formate is then further processed by the formate hydrogen lyase (FHL-1) complex, encoded by the *hyc* operon, producing CO₂, H₂, and different organic acids. This will then lead to an increased ECAR^34,35^, as observed in our experiments. This metabolic shift is supported by our microarray data showing that the *hyc* genes are significantly upregulated (Supplementary Table S1), while the formate transporter *focA* is downregulated. This will likely promote intracellular formate retention and sustained FHL-1 activity. In addition, genes involved in nitrate and nitrite respiration (*napA*, *nirB*, *nirD*) are downregulated, indicating suppression of anaerobic respiration^36,37^.

Next, we also observed an upregulation of the antioxidant defense genes *katG* (catalase-peroxidase), *ahpCF* (alkyl hydroperoxide reductase) and *grxA* (glutaredoxin) in IL-1β-stimulated CFT073. The upregulation of these genes suggests an activation of the OxyR peroxide-sensing regulatory system in CFT073. OxyR is a redox-sensitive transcription factor that responds to hydrogen peroxide and triggers the expression of antioxidant defense genes, especially *katG*, *ahpCF* and *grxA*^38,39^. These enzymes buffer intracellular ROS and restore redox balance. This is essential adaptation during infection, where oxidative stress can arise both from host immune responses and from metabolic shifts such as increased fermentation^38,39^. Enrichment analysis showed that the GO terms; response to reactive oxygen species, cellular response to hydrogen peroxide and cell redox homeostasis were significantly enriched based on our microarray data. This supports an activation of the OxyR peroxide-sensing regulatory system. Furthermore, in the context of UPEC pathogenesis, OxyR plays an important role in promoting bacterial survival during inflammatory conditions^40,41^. The urinary tract is a hostile milieu full of neutrophil-derived ROS and reactive nitrogen species, particularly during IL-1β-driven immune activation^42^. Previous studies have shown that UPEC strains lacking OxyR are significantly impaired in their ability to colonize the bladder, highlighting its importance as a virulence factor^40,43^.

Our microarray analysis also showed significant upregulation of purine biosynthesis genes (*purL*,* purF*,* purH* and *purK*) in response to IL-1β stimulation. This was accompanied by the enrichment of the KEGG “purine metabolism” pathway. De novo purine synthesis is essential for maintaining nucleotide pools required for DNA replication, RNA transcription, and energy transfer. Functions that are important during rapid bacterial growth in nutrient-limited environments like the urinary tract^44^. Studies have shown that purine biosynthesis contributes directly to UPEC fitness and virulence, with *pur* mutants showing impaired bladder colonization^45,46^. Taken together, these findings may suggest that IL-1β stimulation promotes a metabolic and redox state that supports UPEC persistence during immune-mediated stress. Activation of the OxyR regulon enhances oxidative stress tolerance, while increased purine biosynthesis ensures sufficient nucleotide availability to sustain growth and colonization. Together, these responses may contribute to a virulence-enhancing metabolic adaptation that could favour UPEC survival in the urinary tract.

We next investigated whether IL-1β influences the ability of CFT073 to adhere to and invade bladder epithelial cells, based on the upregulation of genes observed in our microarray data. We found that the type-1 fimbriae (*fimH*) and the P-fimbriae (*papC*) were upregulated by IL-1β. We also observed that IL-1β increased CFT073’s ability to colonize (adhesion/invasion) and invade bladder epithelial cells, in a dose dependent way. The ability of UPEC to adhere to and invade uroepithelial cells is a key step in the establishment of UTI. The type-1-fimbriae is primarily involved in bladder colonization, while P-fimbriae facilitate attachment to the kidney epithelium^5,47^. In addition, we also evaluated UPEC motility and found no changes induced by IL-1β. Furthermore, since the release of IL-6, IL-8, and IL-1β play an important role in the host response to UPEC and other bacteria^13,15–18,22,48–51^, we next investigated whether IL-1β-stimulated CFT073 could modulate cytokine release from bladder epithelial cells. We found that IL-1β-stimulated CFT073 did not increase the release of IL-6, IL-8 or IL-1β from bladder epithelial cells. However, additional cytokines and chemokines should be examined in future studies to rule out the possibility that IL-1β-stimulated CFT073 alters their release. Hence, UPECs ability to sense IL-1β and upregulate *fimH* and *papC*, in combination with the increased adhesion and invasion of bladder epithelial cells, may indicate a promoted persistence in the urinary tract.

To further evaluate how IL-1β-induced changes in CFT073 contribute to overall virulence, we continued using a *C. elegans* infection model. This model has previously been validated for assessing UPEC pathogenicity and has shown to correlate well with murine infection outcomes^23,52,53^. We found that *C. elegans* infected with IL-1β-stimulated CFT073 downregulated several genes involved in innate immunity: *abf-2* (antimicrobial peptide), *lys-7* (lysozyme), and *tir-1* (Toll and Interleukin 1 Receptor-1), as well as stress-response genes (*ape-1*, *cep-1*). Each of these genes contributes to nematode defense or stress tolerance^54–56^. Their downregulation indicates that IL-1β-stimulated UPEC actively dampens/suppresses host defense responses in *C. elegans*. Interestingly, these changes did not seem to be dose dependent. UPECs ability to modulate the immune response has been shown to be a key aspect of its virulence in the urinary tract^7–9^. We have previously shown that IL-1β-stimulated CFT073 decreased the survival of *C. elegans*^23^. Our current findings suggest that the suppressed immune response induced by IL-1β-stimulated CFT073 may partly account for this increased lethality.

As antibiotic resistance in UPEC continues to rise, targeting bacterial virulence offers a promising alternative to traditional therapies. Our study shows that IL-1β, a key inflammatory cytokine, enhances UPEC virulence by inducing metabolic and transcriptional changes that promote fermentative growth, oxidative stress resistance, and purine biosynthesis. These adaptations could support bacterial survival under immune pressure and increase adhesion, invasion, and immune suppression in the host. These findings highlight a paradox where host inflammation may unintentionally enhance pathogen fitness. Targeting these host–bacterial interactions may offer new strategies for preventing or managing UPEC infections.

## Supplementary Information

Below is the link to the electronic supplementary material.

Supplementary Material 1

Supplementary Material 2

## Data Availability

All data generated or analysed during this study are included in this published article (and its Supplementary Information files). Gene expression data is available upon request.
