# Supplementary material for: IL-1β alters the virulence of uropathogenic Escherichia coli
Source: Sci Rep. 2025 Oct 29;15:37750. doi: 10.1038/s41598-025-26055-4 (PMC12572196; doi:10.1038/s41598-025-26055-4)
Supplement: Supplementary file 2 — Supplementary Material 2 [file 41598_2025_26055_MOESM2_ESM.docx]

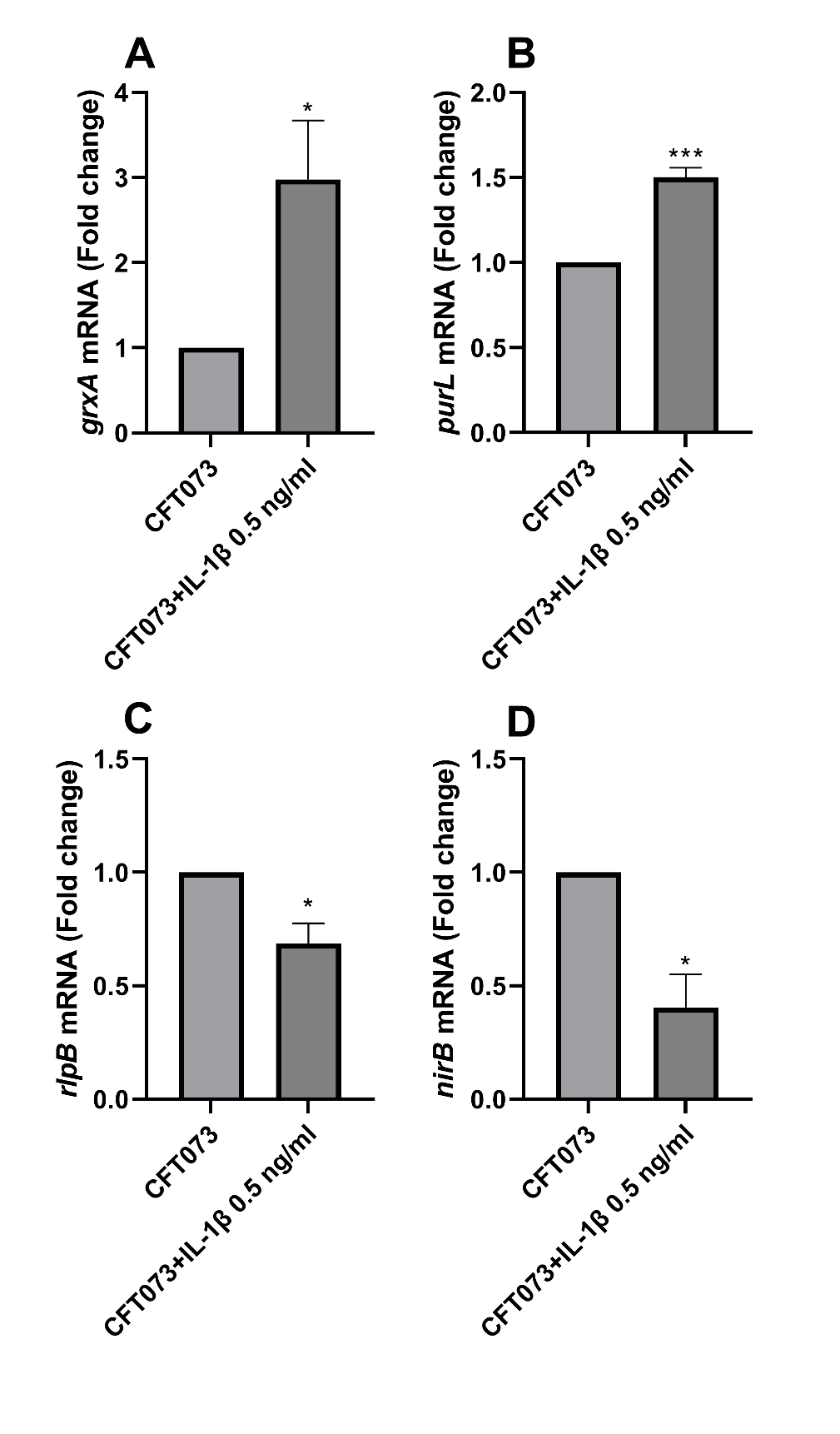


**Figure S1**. Real time-qPCR analysis of *grxA* (**A**), *purL* (**B**), *rlpB* (**C**) and *nirB* (**D**) mRNA expression in the presence or absence of IL-1β (0.5 ng/ml) after 6 hours. Data are presented as mean ± SEM of n=3 independent experiments
